# Preservation of Genes Involved in Sterol Metabolism in Cholesterol Auxotrophs: Facts and Hypotheses

**DOI:** 10.1371/journal.pone.0002883

**Published:** 2008-08-06

**Authors:** Giovanna Vinci, Xuhua Xia, Reiner A. Veitia

**Affiliations:** 1 Institut Cochin, Département de Génétique et Développement. Inserm, U567, CNRS, UMR 8104, Université Paris 5, Faculté de Médecine Paris Descartes, UM 3, Paris, France; 2 CAREG and Biology Department, University of Ottawa, Ottawa, Ontario, Canada; 3 Université Denis Diderot/Paris VII, Paris, France; Ecole Normale Supérieure de Lyon, France

## Abstract

**Background:**

It is known that primary sequences of enzymes involved in sterol biosynthesis are well conserved in organisms that produce sterols *de novo*. However, we provide evidence for a preservation of the corresponding genes in two animals unable to synthesize cholesterol (auxotrophs): *Drosophila melanogaster* and *Caenorhabditis elegans*.

**Principal Findings:**

We have been able to detect *bona fide* orthologs of several *ERG* genes in both organisms using a series of complementary approaches. We have detected strong sequence divergence between the orthologs of the nematode and of the fruitfly; they are also very divergent with respect to the orthologs in organisms able to synthesize sterols *de novo* (prototrophs). Interestingly, the orthologs in both the nematode and the fruitfly are still under selective pressure. It is possible that these genes, which are not involved in cholesterol synthesis anymore, have been recruited to perform different new functions. We propose a more parsimonious way to explain their accelerated evolution and subsequent stabilization. The products of *ERG* genes in prototrophs might be involved in several biological roles, in addition to sterol synthesis. In the case of the nematode and the fruitfly, the relevant genes would have lost their ancestral function in cholesterogenesis but would have retained the other function(s), which keep them under pressure.

**Conclusions:**

By exploiting microarray data we have noticed a strong expressional correlation between the orthologs of *ERG24* and *ERG25* in *D. melanogaster* and genes encoding factors involved in intracellular protein trafficking and folding and with *Start1* involved in ecdysteroid synthesis. These potential functional connections are worth being explored not only in *Drosophila*, but also in *Caenorhabditis* as well as in sterol prototrophs.

## Introduction

Cholesterol and other sterols such as ergosterol and phytosterols are universal components of eukaryotic plasma membranes and are absent from the membranes of prokaryotes. These sterols (cholesterol, ergosterol and phytosterol) modulate membrane fluidity [1], [2]. In addition to this structural role, cholesterol is essential for signaling processes. In fact, it is a precursor of steroid hormones, oxysterols, ecdysones (in insects) and vitamin D. Moreover, it may influence intercellular signaling through its covalent attachment to proteins such as the protein Hedgehog (Hh) in *Drosophila* [3]–[5]. The nematode *Caenorhabditis elegans* possesses several genes encoding proteins with regions similar to Hh and potentially undergoing cholesterylation [6], [7].

**Figure 1:**
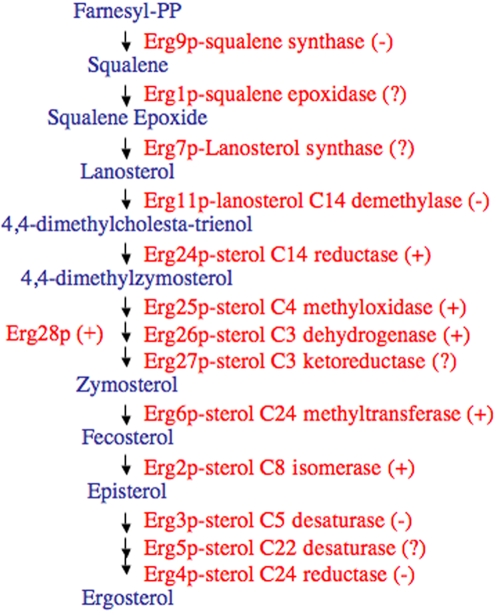
Outline of the ergosterol synthesis pathway in yeast. (+) the corresponding gene is present in *C. elegans* and *D. melanogaster*, according to our exploration. (−) the corresponding gene is absent. (?) not convincing evidence for the presence of the ortholog.

Yeast, plants and mammals synthesize sterols through a series of complex reactions that occur in the endoplasmic reticulum (ER) and, therefore, most of the involved enzymes have transmembrane domains (Figure 1). We outline below the series of reactions to produce ergosterol in the yeast *Saccharomyces cerevisiae*, which involve *ERG* (i.e. from ERGosterol) genes and the respective ERG proteins.

The first three steps of sterol biosynthesis are catalized by ERG9p (squalene synthase), ERG1p (squalene epoxidase) and ERG7p (lanosterol synthase), respectively. These proteins are essential for aerobic viability and their absence results in an inability to synthesize ergosterol. The third enzyme, ERG7p, converts squalene epoxide into lanosterol, the first cyclic component of the cholesterol biosynthesis cascade (i.e. the first sterol). The remaining enzymes of the pathway metabolize lanosterol into ergosterol. The sequential action of ERG11p (lanosterol demethylase) and ERG24p (C-14 reductase) leads to 4,4-dimethylzymosterol. They are also essential in aerobic conditions [8]–[10]. Removal of the two C-4 methyl groups of 4,4-dimethylzymosterol is a complex reaction involving the products of genes *ERG25*, *ERG26*, and *ERG27*, in cooperation with ERG28p. Finally, ERG6p (C-24 methylase) converts zymosterol to fecosterol, which is further transformed into ergosterol by ERG2p, ERG3p, ERG5p and ERG4p [8]–[10] (Figure 1).

Recent results show that, in yeast, ergosterol biosynthetic enzymes display specific protein-protein interactions and form a functional complex called ergosome. Proteins ERG11p, ERG25p, ERG27p and ERG28p, appear to form a core center of the complex and would interact with other enzymes of the pathway [9], [10]. Indeed, the small transmembrane protein ERG28p functions as a scaffold to tether the C-4 demethylation complex (ERG25p-ERG26p-ERG27p) to the ER [11] and also interacts with the downstream enzyme ERG6p. More recent results have indicated that ERG28p also interacts with ERG11p and ERG1p. Moreover, ERG27p is required for oxidosqualene cyclase (ERG7p) activity. The physical interaction of ERG27p with ERG7p might indeed contribute to yeast sterol biosynthesis regulation [12]. These results altogether suggest that many sterol biosynthetic proteins, if not all, may be tethered to the ER as a large complex [9], [10].

Most animals synthesize cholesterol. However, some animals such as *D. melanogaster* and *C. elegans* cannot synthesize sterols *de novo* [13], [14]. They are auxotrophic for sterols because they do not possess the enzymatic activities necessary to complete this process [13], [14]. *C. elegans* takes sterols from animal feces or yeast and plant remnants [3]. *Drosophila* obtains sterols from the diet: ergosterol from yeast and phytosterols from plants [15]. However, both animals express the homologs of the enzymes that produce the initial intermediates of sterol biosynthesis up to the very early precursor farnesyl pyrophosphate. However, they cannot synthesize either squalene or lanosterol, key intermediates of sterol biosynthesis [13], [14].

As already noted, sterols in these animals are required as hormone precursors and/or developmental effectors [16]. Not surprisingly, *dauer larva* formation and molting depend on sterols. Cholesterol, or more likely its derivatives, seem to act as hormones. Indeed, recent papers report the identification of natural steroid ligands for the DAF-12 nuclear receptor [17].

In *C. elegans*, the distribution and transport of cholesterol *in vivo* has been studied by using dehydroergosterol (DHE), a fluorescent analog which mimics many cholesterol properties [18], [19]. DHE accumulates primarily in the pharynx, nerve ring, excretory gland cell, and gut of L1–L3 larvae [20]. Interestingly, sterols present in the pharynx and in the intestine of feeding animals are distributed in a proximal-to-distal gradient. Cholesterol in *C. elegans* might be involved in the structural and functional organization of the plasma membrane cell types that are richer in this lipid [21] and in modulating the activity of signaling molecules (such as Hh-like proteins).

A previous comparative genome analysis of *D. melanogaster* with *Anopheles gambiae* and prototrophs has suggested that these insects have lost most of the genes involved in sterol synthesis [22]. This makes sense, knowing that *Drosophila* is unable to synthesize cholesterol. However, in a previous work we have shown that *Drosophila* contains an *ERG28* ortholog that has undergone a process of acceleration in its evolution, and is undetectable using the current techniques for ortholog detection by sequence homology [23]. Thus, here we have revisited this question for both *C. elegans* and *D. melanogaster*, not only for *ERG28*, but most of the genes/enzymes involved in the sterol synthesis pathway, in the light of new genomic and functional data.

## Results and Discussion

### Looking for *ERG* orthologs in *C. elegans* and *D. melanogaster*

**Figure 2:**
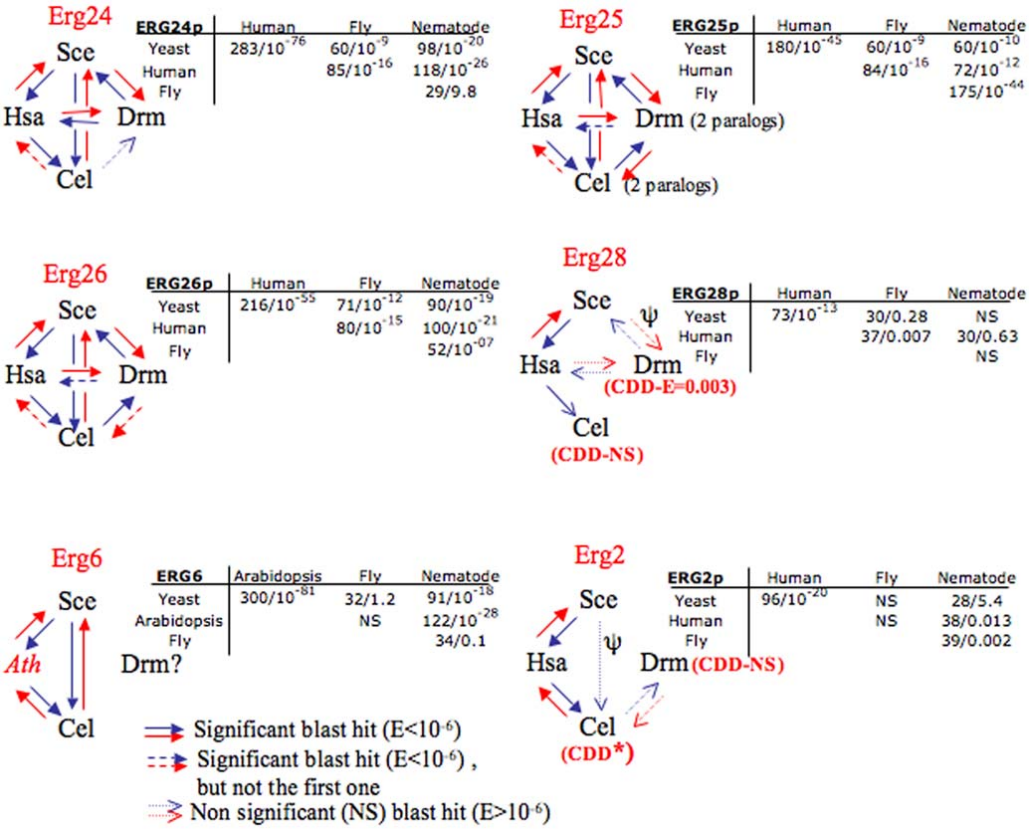
Details of the BLAST analysis that allowed the detection of *ERG* orthologs in *C. elegans* and *D. melanogaster*. The comparisons were performed in the directions indicated by the arrows (i.e. Yeast→Human represents a BLASTp search with an ERG protein from yeast in the division *Homo sapiens* of Genbank). Low Scores S and high E-values (>10^−6^) are classically considered as non-significant (unrelated or divergent sequences). The small tables display the S and E-values for the comparisons using the species-specific divisions of Genbank.

In this section we present our search for *ERG* gene orthologs in *C. elegans* and *D. melanogaster* following their order in the sterol synthesis cascade (as shown in Figure 1). We have taken advantage of the fact that the full genomic sequences of these two animals are available. We have used BLASTp [24] and considered as orthologs the best reciprocal hits [25]. The results are summarized in Figures 1 and 2.

We failed to detect the squalene synthase (ERG9p) homolog, which catalyzes the first committed step in cholesterogenesis. In the search for squalene epoxidase (ERG1p) in a BLAST using ERG1p from yeast, we detected the gene CBG19254 in *C. briggsae*, with a marginal Score (S = 48 bits, E-value = 10^−04^). This protein, which also exists in *C. elegans*, contains two functional domains: monooxygenase and UbiH (2-polyprenyl-6-methoxyphenol hydroxylase and FAD-dependent oxidase). This is also the case in yeast ERG1p. However, in a reverse BLAST, CBG19254 recognized with very high score yeast Coq6 and only marginally ERG1p (S = 48, E = 10^−06^). A similar behavior was observed for GA20231-RA from *D. pseudoobscura*, so we did not proceed to a further analysis of these sequences.

For lanosterol synthase (ERG7p), which follows in the classical pathway, we could not gather any convincing evidence for the existence of orthologs in the fruitfly and the nematode.

The following enzyme in the pathway is a sterol 14α-demethylase, ERG11p/Cyp51, involved in the biosynthesis of cholesterol, phytosterols and ergosterol. Thus, it is the only cytochrome P450 having an ortholog common to animals, plant and fungi [26]. Similarity BLAST hits with yeast ERG11p/Cyp51, were obtained in *Drosophila* (CG2397, CG10247 and other Cyps). However, the *Drosophila* genes are likely to belong to other Cyp subfamilies (not Cyp51). Cyp51 is probably missing, which is in agreement with the results of Tijet, Helvig & Feyereisen [26] who analyzed 90 sequences of the cytochrome *P450* gene superfamily. Cyp51 is also absent in *C. elegans* [26].

Potential *ERG24* orthologs in *D. melanogaster* (CG17952) and in *C. elegans* (B0250.9) were easily found. The corresponding proteins contain the ERG4-24 domain. *D. melanogaster* produces three isoforms that are longer than the yeast ortholog, a peculiarity that they share with the human ortholog. The ortholog of *ERG24* in mammals encodes the Lamin B receptor (LBR), a nuclear envelope protein first described in vertebrates. LBR bears extensive structural similarities with the members of the sterol reductase family (ERG24p and ERG4p). Human LBR (hLBR) cannot restore ergosterol biosynthesis in an *ERG4* yeast mutant, whereas it is able to restore ergosterol prototrophy in an *ERG24* mutant. This strongly suggests that hLBR is a sterol C14-reductase [27]. Not surprisingly, a mutation in the *hLBR* gene causes an autosomal recessive disease called hydrops-ectopic calcification-‘moth-eaten’ (HEM). This mutation leads to high levels of cholesta-8,14-dien-3-beta-ol in cultured skin fibroblasts, which is compatible with a deficiency of the cholesterol biosynthetic enzyme 3-beta-hydroxysterol delta(14)-reductase [28].

The hLBR contains two major domains: a ∼220-amino-acid N-terminal segment highly charged, and a hydrophobic C-terminal half with eight putative transmembrane segments [29], [30]. Interestingly, it has been hypothesized that the region encoding the N-terminal domain of the *LBR* gene arose from an ancestral gene coding for a soluble nuclear protein (which provides a nuclear localization signal) and that the rest of the protein evolved from another gene, similar to yeast *ERG24*. Indeed, the C-terminal hydrophobic domain of LBR can be retained in the endoplasmic reticulum when expressed in transfected cells, as expected for the ortholog of *ERG24* in mammals. In turn, the N-terminal domain is transported to the nucleus [31]. This domain might be responsible for the targeting of the hydrophobic domain to the nucleus and for the interaction with lamin B [32]. So far, cholesterol synthesis is supposed to occur in the smooth ER. Since the N-terminal domain of LBR is responsible for its nuclear localization, it would be interesting to investigate whether the LBR transcript, or protein, undergoes some processing leading to the production of a C-terminal domain sorted to the ER.

Recent functional studies show that the *Drosophila* CG17952 gene is the ortholog of vertebrate *LBR* [33]. The protein encoded by CG17952 shares some properties with hLBR. The *Drosophila* LBR (dLBR) possesses a highly charged N-terminal domain of 307 amino acids followed by eight transmembrane segments. Transmembrane segments 1–6 are similar in length and position to the transmembrane domains 1–6 of hLBR. However, the putative membrane domains 7 and 8 of dLBR are shorter than those of hLBR. Thus, dLBR is expected to have a topological organization similar to that of its vertebrate orthologs [33]. dLBR is able to bind to the *Drosophila* lamin B, a function residing in the N-terminal domain. Not unexpectedly, dLBR does not display sterol C14 reductase activity when expressed in the yeast *ERG24* mutant. This shows that, during insect evolution, although the enzymatic activity of this protein has been lost, its capacity to bind lamin B has not. However, depletion of dLBR by RNA interference does not lead to any obvious effect on nuclear architecture, or viability, of treated cells and embryos. Thus, although dLBR might be important, it is not a limiting component of the nuclear architecture in *Drosophila* cells, at least during the first days of development [33]. Our BLAST search shows that sequence B0250.9 is the potential *ERG24* ortholog in *C. elegans*. It would be interesting to experimentally assess if it has kept LBR activity.

**Figure 3:**
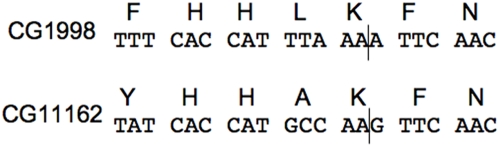
Segments of the *Drosophila* paralogs CG1998 and CG11162 (homologs of Erg25) and the corresponding conceptual translations. The interruption of the open reading frames of both genes, by their last intron, is shown by vertical lines.

Sequences homologous to ERG25p (C-4 methyloxidase) were also easily found in both *D. melanogaster* (CG1998/*dERG25A* and CG11162/*dERG25B*) and *C. elegans* (F49E12.9/*ERG25A* and F49E12.10*/ERG25B*) using the sequence of yeast ERG25p as a starting point. In both organisms the duplicated copies of *ERG25* are located in the same chromosome (chromosome II for *C. elegans* and chromosome X for *D. melanogaster*). The two paralogs in *D. melanogaster* are separated by 0.25 Mb and contain a different number of predicted exons. Namely, CG1998 contain 6 exons, while CG11162 contains only 2. However, the last intron of both genes interrupts the coding sequence at very similar positions (i.e. between the second and the third positions of a Lys codon, Figure 3). In *C. elegans* the paralogs are at less than 3 kb away from each other. F49E12.9 contains 8 exons while F49E12.10 has 5 exons that may have been produced through exon fusion/splitting events.

ERG26p (C-3 dehydrogenase) belongs to the 3β-hydroxysteroid dehydrogenase family and convincing evidence for the presence of orthologs in *C. elegans* (ZC8.1) and *D. melanogaster* (CG7724) was obtained in BLAST searches taking the sequence from yeast as the starting point (See details in Figure 2). In the case of *Drosophila* CG7724, similarity with yeast ERG26p extends over the first 250 amino acids while the remaining amino acids are more divergent. Interestingly, when performing a BLAST with the *Drosophila* sequence, the first hit in *C. elegans* was C32D5.12, but it was not the best hit either with yeast, or with *A. thaliana*, or even with the human (NSDHL) orthologs. However, a BLAST with C32D5.12 detected as the first hits NSDHL in man and ERG26 in yeast (Figure 2). Thus, it is tempting to invoke some kind of sequence convergence as an explanation for this behavior.

In agreement with Breitling *et al.* [34] we failed to find any clear homologue of ERG27p (C-3 ketoreductase) in both *Drosophila* and *C. elegans*, although several oxidoreductases were detected.

**Figure 4:**
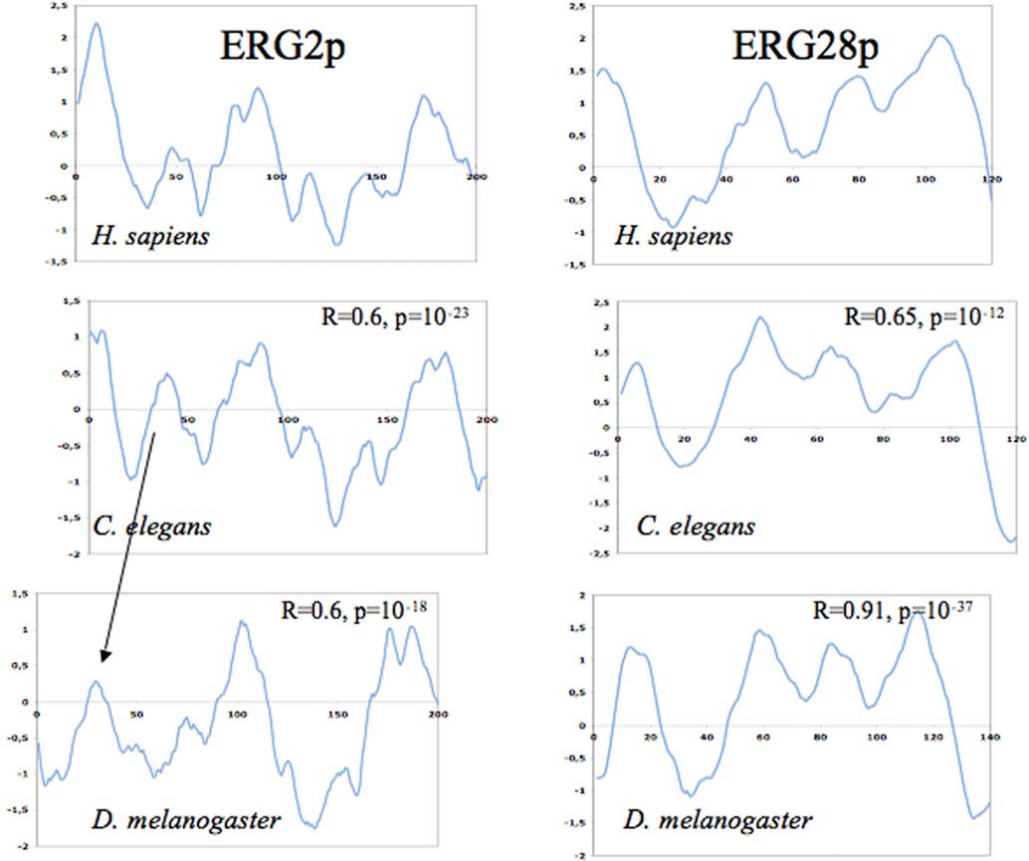
Hydrophobic profiles of several potential ERG orthologous proteins. A way to show a structural relationship is to predict the hydrophobic profiles of the relevant proteins. Here we have used the TopPred program [36] as implemented in the server of Pasteur Institute (http://www.pasteur.fr). Left panels show the results for the potential homologs of ERG2p while the right panels display the profiles for ERG28p homologs (using the Kyte-Doolitle scale, with the default parameters). Negative (positive) values represent hydrophobic (hydrophilic) segments. A way to statistically assess the similarity of two profiles is to calculate their correlation coefficient R. R-values for pairwise comparisons with the human sequence are reported. We tried to maximize the R-value by slightly sliding one profile over the other (that is why the frames of the profiles are not perfectly aligned).

As outlined above, ERG28p might tether many other ERG proteins to the ER. The ERG28p ortholog of *C. elegans* (C14C10.6) was hardly detectable by BLAST starting with yeast sequences. This precludes the use of standard phylogenetics methods to show orthology. However, further evidence of sequence relatedness was gathered using Psi-BLAST with the yeast sequence against the Metazoa division of Genbank [35]. We included in the iterations the very divergent sequence CG17270 of *D. melanogaster*, which is the ortholog of ERG28p according to our previous results [23]. This allowed us to detect C14C10.6 as a signinficant hit (S = 120 bits and E = 10^−27^). Reverse Psi-BLAST also suggested orthology (i.e. significant scores). The sequence of C14C10.6 is so divergent that it had no match in the conserved domain database (CDD). Moreover, we also computed the hydrophobic profiles for some of the orthologs [36] and calculated the Pearson correlation coefficient “R” for various pairs of profiles. Significant R-values were obtained for the pair-wise comparisons (Figure 4). This is not a proof of orthology but strengthens the idea of structural relationship at the protein level. Finally, we found that all proteins had similar lengths and were basic, with isoelectric points (pI) >8.5.

The ortholog of ERG6p in *C. elegans* (H14E04.1) was easily detected by BLAST with protein sequences from either yeast or *A. thaliana* (SMT1). We used the plant sequence since no clear *ERG6* homolog could be detected in human. The issue with *Drosophila* turned out to be more complex because, when starting the search with the yeast sequence, we detected CG8067 marginally (E>1). This was even worse when starting with the sequence of *A. thaliana*. However, when using the *C. elegans* sequence as a starting point, the first significant hit in *Drosophila* (E<10^−6^) was CG2453, which proved to be the ortholog of yeast Coq5, but not of ERG6p. Finally, considering i) the similar lengths of the previously marginally detected CG8067, of H14E04.1 and of SMT1 proteins and ii) their similar pI, we have preferred CG8067 as the most likely ortholog. Indeed, the hydrophobic profiles of SMT1 and the protein encoded by CG8067 displayed a strong correlation. Namely, we obtained an R = 0.43 with a p-value 10^−16^.

The ortholog of ERG2p in *C. elegans* (W08F4.3) was marginally detected by BLAST with the yeast and the human protein (opioid sigma-1 receptor, OPRS1). However, W08F4.3 was found to contain a sigma1-receptor domain when compared with the CDD. This strengthens the idea that this gene is the ortholog of *OPRS1* and *ERG2*. The situation in *Drosophila* was more complex. When starting our BLAST with either yeast or human sequences, we detected the sequence HDC14735 (DAA04220) very marginally (E≫1) and no conserved domain was found. However, when starting with the *C. elegans* sequence, it came as the best hit with E = 0.002 (the reverse was also true, with E = 0.003). Although not significant, this result was taken as suggestive of similarity. The results were improved using Psi-BLAST. Again, we computed the hydrophobic profiles for the various potential orthologs and we found strong correlations (Figure 4). Finally, the pI of the protein encoded by HDC14735 (pI = 6.14) was comparable to those of OPRS1 (5.61) and ERG2 (5.54) (see below).

In BLAST searches with yeast ERG3p, we detected again CG1998 and CG11162 in *D. melanogaster* and F49E12.9 and F49E12.10 in *C. elegans*, but with worse scores than in BLASTs with ERG25 (S∼55 bits versus 85 bits respectively). Therefore, we propose that the orthologs of ERG3 are potentially missing in both organisms.

For ERG5p, which belongs to the big Cyp protein family, no clear orthologs could be established. However, three potential candidates were found: CG4321-PA (Cyp4d8), CG3540-PA (Cyp4d14) and CG8859-PA (Cyp6g2). In the reverse BLAST, they all matched ERG5p as the best scoring hit in yeast.

Finally, the search for ERG4p orthologs led to the same ERG24 orthologs in the nematode and the fruitfly. Moreover, with ERG4p the BLAST scores were worse than with ERG24p. Thus, either ERG4 orthologs are missing or they have been replaced by ERG24.

### Evolution of the *ERG* orthologs in *C. elegans* and *D. melanogaster*

To assess whether some of the *ERG* orthologs mentioned above undergo a selective pressure, we have examined the ratio of the number of non-synonymous substitutions per non-synonymous site (Ka) and the number of synonymous substitutions per synonymous site (Ks). The ratio Ka/Ks is indicative of the mode of evolution operating on the sequences. If selection is dominantly purifying, then we expect few non-synonymous substitutions per background synonymous changes and hence, a low ratio. If selection is absent, then a ratio of unity is expected (Table 1). To obtain the Ka/Ks values for the fly we compared the sequences from *D. melanogaster* and *D. pseudoobscura* while for the nematode we compared the sequences from *C. elegans* and *C. briggsae*. In spite of the divergence of the various *ERG* orthologs in the lineages leading to insects and to nematodes, the low value of Ka/Ks (dN/dS) ratios show that these genes are under selective pressure (Table 1).

**Table 1:** Ka/Ks and dN/dS values for several orthologs of *ERG* genes in the fruitfly and the worm.

Orthologs	*C. elegans* vs *C. briggsae*		*D. melanogaster* vs. *D. pseudobscura*	
*ERG*	Ka/Ks	dN/dS	Ka/Ks	dN/dS
2	0.127	0.100	0.080	0.029
6	0.044	0.029	0.041	0.016
24	0.055	0.055	0.122	0.085
25A	0.041	0.039	0.064	0.041
25B	0.055	0.031	0.138	0.087
28	0.080	0.064	0.033	0.013

It is natural to ask what protein properties have been conserved by the purifying selection. We have investigated a protein-level property, namely, the isoelectric point (pI). This property is important for enzymes because protein-protein and enzyme-substrate interactions are often electrostatic in nature. Thus, we should expect the pI of an enzyme to be similar in different organisms if protein-protein and/or enzyme-substrate interactions are conserved. In order to test this idea we gathered the protein sequences of the orthologs of ERG2, 6, 24, 25, 26 and 28. Next, we asked whether the corresponding *Drosophila* and *Caenorhabditis* sequences displayed outlier pI values. None of the orthologous proteins in the auxotrophs was detected as outlier, suggesting conservation of this physico-chemical property within the nematode and the fly lineages in spite of primary sequence divergence (Table 2). Similarity in pI is expected, when the orthologs are detected as the best reciprocal significant hits in BLAST searches. However, the existence of similarity in the hydrophobic profiles and the pI between the potential orthologs, for which the BLAST comparisons failed to be significant, is more surprising. It means that, in spite of important sequence divergence, which renders BLAST unreliable in many cases, the proteins have maintained substantial structural and physico-chemical similarity.

**Table 2:** Isoelectric points (pI) of the ERGp orthologs in *C. elegans* and *D. melanogaster*.

Proteins (n)	*C. elegans*	*D. melanogaster*	pI (w/o) mean+/−std	pI (w) mean+/−std
**Erg6 (7)**	5.95 (H14E04.1)	5.79 (CG8067)	5.98+/−0.38	6.19+/−0.83
**Erg26 (16)**	6.52 (ZC8.1)	8.99 (CG7724)	7.93+/−1.23	7.91+/−1.22
**Erg24 (13)**	9.38 (B0250.9)	9.89 (CG17952)	9.19+/−0.67	9.25+/−0.64
**Erg28 (4)**	9.02 (C14C10.6)	9.62 (CG17270)	9.31+/−0.39	9.31+/−0.35
**Erg2 (12)**	9.39 (W08F4.3)	5.49 (HDC14735)	6.28+/−1.02	6.44+/−1.28
**Erg25 (15)**	7.57 (F49E12.9/25A)	8.17 (CG1998/25A)	7.83+/−0.63	7.96+/−0.73
	8.4 (F49E12.10/25B)	9.76 (CG11162/25B)		

The mean pI values (and standard deviations –std) of the orthologous proteins in cholesterol prototrophs are shown for comparison. “pI (w/o)” stands for means +/− std calculated **w**ith**o**ut taking into account the orthologs in *C. elegans* and *D. melanogaster* while “pI (w)” stands for means +/− std calculated including the orthologs in *C. elegans* and *D. melanogaster*. “n” is the number of orthologs in cholesterol prototrophs used to calculate the means.

### Analyzing expression-profiling data to explore the potential function to the divergent *ERG* orthologs

**Figure 5:**
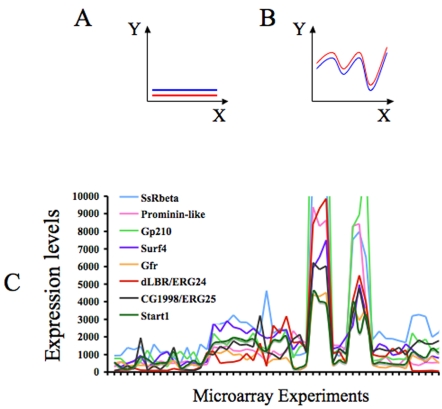
Expression profiles of several genes expressionally correlated with dLBR and CG1998. Panels A and B show schematic profiles displaying very strong R. However, in panel A “co-variation” is not very informative, in this case either the red and blue genes are not expressed or do not change their expression in the conditions analyzed. To avoid this artificial inflation of R, we have focused on experiments where the genes of interest display strong variation (as in panel B). Panel C: Expression profiles of several genes involved in protein trafficking and folding, which co-vary with the expression of dLBR and CG1998. The profile of Start1 is also represented.

Co-expression is indicative of i) physical interaction between proteins and ii) of membership to the same complex or molecular process [37]. This is called the paradigm of “guilt by association” [38]. Thus, we have used published microarray expression data, downloadable from the Gene Expression Omnibus of the NCBI, to investigate potential co-expression patterns of the *ERG* orthologs that we have described above for both *D. melanogaster* and *C. elegans*. Co-expression can be assessed by determining the correlation coefficient. The correlation coefficient can be artificially inflated by flat profiles (no changes in the expression of the relevant genes). To avoid this, we focused on experiments where the genes of interest display strong variation (see Figure 5A and B). Thus, we gathered data concerning 51 different microarray experiments for *D. melanogaster* respecting the criterion outlined previously. We performed a similar analysis for *C. elegans* but, unfortunately, the most interesting genes showed flat profiles (close to 0 in all experiments) and it was not possible to proceed further with the analysis.

First, we asked whether the expression profiles of the *D. melanogaster ERG* orthologs were correlated (Table 3). The strongest correlation was found between the *dLBR* (*ERG24*) and CG1998 (*ERG25*) with an associated p-value of 10^−16^ (after a Bonferroni correction). Such a p-value means that only one correlation coefficient out of 10^16^ is expected to be as high as 0.89 just by chance (for n = 51 experimental points). Considering the maximum number of possible correlations for the 14000 transcripts in the microarrays (representing the *Drosophila* genome), such a high R cannot be found by chance. The behavior of dLBR and CG1998 might be reminiscent of the situation in yeast because ERG24p and ERG25p are supposed to interact, according to Epistatic MiniArray Profiling experiments [39]. CG7724 and CG11162 displayed the second highest R (R = 0.4, p = 0.03), but this R is not relevant in genomic terms.

**Table 3:** Expressional correlation among *D. melanogaster ERG* orthologs.

	CG1998 ERG25A	CG11162 ERG25B	LBR ERG24	CG17270 ERG28	CG8067 ERG6	CG2453 ERG6?
**CG7724**	0.22	0.40	0.18	0.14	0.32	0.27
**CG1998**		0.03	0.89	−0.08	0.01	0.14
**CG11162**			0.07	0.27	0.28	0.34
**LBR**				−0.06	−0.02	0.17
**CG17270**					0.31	0.41
**CG8067**						0.55

The analyses were performed using data downloaded from the Gene expression-Omnibus database (http://www.ncbi.nlm.nih.gov/entrez/query.fcgiCMDsearchDBgeo). We considered the datasets GDS192 (wing imaginal disc spatial gene expression), GDS653 (neurotransmitter-specific neuronal gene expression), GDS664 (splicing factor mutant at permissive and restrictive temperatures) and GDS667 (mRNA splicing factor knock-down) which contain 51 data points for 14 000 transcripts.

At first, we were expecting good expressional correlation among the *ERG* orthologs in *Drosophila*. However, it seems that only dLBR and CG1998 still “remember” their ancestral belonging to the sterol biosynthesis pathway. Poor expressional correlation among the rest of the *ERG* orthologs also suggests that the corresponding proteins either have lost their ability to physically interact in order to form stable complexes, or they do so in conditions/moments not covered by the microarray experiments explored here. Then, we focused our attention on *dLBR* and CG1998 by determining which other genes were expressionally correlated with them. For this, we gathered 84 genes displaying R≥0.875 with respect to both genes. For a correlation involving 51 data points, this R cut-off is associated with a safe p-value of 10^−13^ after correction (Figure 5C). In order to get insights about these 84 genes, we used the functional classification tool of the DAVID database (http://david.abcc.ncifcrf.gov/ [40]). This software provides a rapid means to organize large lists of genes into functionally related groups and to unravel biological relationships.

In the analysis using DAVID, the most overrepresented class included genes encoding membrane proteins, often targeted to the ER, where sterol biosynthesis takes place in prototrophs (Group 1, in Table 4). Interestingly, several of these proteins are supposed to be involved in co-translational protein targeting to membranes, signal peptide recognition, heat shock protein-binding, as well as unfolded protein binding, or to be elements of the translocon (a complex of proteins associated with the translocation of nascent polypeptides into the ER [41]). The following functional category (Group 2) contained four transporter proteins while the last functional group involved chaperones (i.e. peptidyl-prolyl *cis-trans* isomerase), chaperone cofactors or unfolded protein-binding factors. A similar analysis conducted using g∶profiler [42] confirmed that genes encoding protein folding actors were overrepresented among the genes displaying strong expressional correlation with dLBR and CG1998 (p<10^−5^). The existence of expressional correlation does not imply any causality. In fact, from this exploration it is not possible to determine whether dLBR and CG1998 somehow interact with other partners to participate in intracellular protein trafficking or folding, or on the contrary, they undergo the action of the latter. Since dLBR has been shown to be a nuclear protein [31], [32], it would be interesting to investigate whether the dLBR transcript or protein are somehow processed to produce a C-terminal polypeptide that might be sorted to the ER. That would explain the expressional correlation between dLBR and ER proteins observed above. On the other hand, we have also explored the annotation of the genes expressionally correlated with the rest of the *ERG* orthologs. However, no unifying theme emerged from this analysis (data not shown, available upon request). All in all, the strong expressional correlation between dLBR and CG1998 with proteins involved in intracellular protein trafficking or folding, and the absence of such correlation with other Erg orthologs (that also require chaperons) suggest that the involvement of dLBR and CG1998 in both processes is worth exploring.

**Table 4:** Functional clustering of genes whose expression profiles stronlgy correlate with those of dLBR and CG1998 (using the DAVID classification tool at Medium stringency).

Gene Group 1	Enrichment Score: 4.16	Gene Name	Key words
1		**CG5885**	cotranslational protein targeting to membrane, integral to ER membrane, SRP, translocon complex
2		**SURF4**	surfeit 4, receptor signaling protein activity, asymmetric protein localization, ER membrane
3		**CG8583**	HSP binding, SRP binding, unfolded protein binding, SRP receptor complex
4		**SEC61ALPHA**	protein translocase activity, SRP-dependent cotranslational protein targeting to membrane, translocon complex
5		**CG32700**	oxidoreductase activity
6		**CG33162**	SRP receptor
7		**GP210**	transporter activity, protein targeting, integral to membrane
8		**SSRBETA**	signal sequence receptor
9		**PROMININ-LIKE**	prominin-like protein, intracellular protein transport, integral to membrane,
10		**CG33105**	intracellular protein transport, Golgi apparatus, integral to membrane
11		**CG1967**	intracellular transporter activity, post-Golgi vesicle-mediated transport, coated vesicle,integral to membrane
12		**CG11857**	vesicle-mediated transport
13		**GFR**	GDP-fucose transport, Golgi-associated vesicle
14		**CG1751**	Probable signal peptidase complex subunit 2
15		**CG33214**	FGF binding, receptor binding, cell adhesion, intracellular protein transport
**Gene Group 2**	Enrichment Score: 1.84		
1		**CG5594**	amino acid-polyamine transporter activity, cation transporter activity
2		**CG15094**	high affinity inorganic phosphate∶sodium symporter activity,serine-type endopeptidase inhibitor activity
3		**CG8291**	neurotransmitter transporter activity
4		**CG6293**	L-ascorbate∶sodium symporter activity, nucleotide and nucleic acid transport
**Gene Group 3**	Enrichment Score: 1.64		
1		**TORP4A**	torsin-like protein precursor, unfolded protein binding, protein folding
2		**CG7872**	HSP binding
3		**FKBP13**	peptidyl-prolyl cis-trans isomerase activity, protein folding, ER
4		**CG14715**	peptidyl-prolyl cis-trans isomerase activity, protein folding, ER
5		**GP93**	glycoprotein 93, unfolded protein binding, response to stress, ER

ER: endoplasmic reticulum, HSP: heat shock protein, SRP: signal recognition particle. At high stringency, only the first functional cluster is obtained.

In a previous paper, given the structural similarity between cholesterol and ecdysteroids, we had proposed that divergent ERGp orthologs might somehow participate in the synthesis of the latter [23]. We have therefore assessed the expressional correlation between candidate genes involved in this process: *Dare1*/CG12390 [43], *Jhamt* [44] and *Start1* [45], with *dLBR* and *CG1998*. While for *Jahmt* and *Dare1* the values of R are below 0.6, a very strong correlation (R = 0.8) was found for *Start1*. Interestingly, *Start1*, which is involved in intra-mitochondrial sterol transport, is expressed ubiquitously. However, *in situ* hybridization demonstrates a stronger expression in the prothoracic gland, where ecdysteroids are synthesized from cholesterol. These and other observations are consistent with the idea that Start1 plays a key role in the regulation of ecdysteroid synthesis [45]. The potential functional link between dLBR, CG1998 and Start1 is also worth exploring.

In conclusion, we detected a preservation of *ERG* genes in *Drosophila melanogaster* and *Caenorhabditis elegans*. In spite of their sequence divergence with respect to the corresponding orthologs in sterol prototrophs, they still are under selective pressure. Since insects are unable to synthesize cholesterol *de novo*, an appealing way to explain this evolutionary acceleration is that ERGp orthologs have other biological functions in addition to sterol synthesis. This is clearly the case of the LBR, which is also a reductase in sterol prototrophs. Shut-down of cholesterogenesis in insects and nematodes would have allowed these proteins to evolve as much as their other functions were not compromised [23]. Another, less parsimonious, explanation would be the evolution of different novel functions. Our microarray meta-analysis shows strong expressional correlation between the orthologs of *ERG24* and *ERG25* in *D. melanogaster* and genes encoding factors involved in intracellular protein trafficking and folding. This is compatible with our idea that ERGp might be involved in other biological roles in addition to sterol synthesis. The potential link between ERG proteins and intracellular protein trafficking and folding deserves experimental exploration not only in *Drosophila* and *Caenorhabditis* but also in sterol prototrophs. Moreover, the potential link between dLBR, CG1998 and Start1 is to be explored in *D. melanogaster*. This is compatible with our previous idea of a potential implication of these proteins in the synthesis of ecdysteroids. We hope that this genomic exploration and the hypotheses prompted here might open new avenues of experimental research.

## Materials and Methods

### Ortholog search

We used BLASTp [24] and considered as orthologs the best reciprocal hits. In short, a BLAST search is performed starting with a protein sequence from organism A to detect the matching sequences in organism B. Then, the sequence from B displaying the highest score is compared (reverse BLAST) against the all sequences of A and the highest scoring hit must be the initial sequence. This is a widely accepted criterion of orthology [25]. We also exploited the significance of matches using Psi-BLAST [35] or with the conserved domain database (CDD), where the scoring of the protein alignment uses very sensitive “tailor made” scoring matrices.

### Computation of hydrophobic profiles as further evidence of structural relationship between potentially orthologous proteins

For some distant homologues we computed the hydrophobic profiles and calculated the Pearson correlation coefficient “R” for various pairs of profiles. The values of R range between 1 (perfect match between the profiles) and −1 (profiles are mirror images). Strong positive correlation is not a proof of orthology but strengthens the idea of structural relationship at the protein level.

### Calculation of Ka/Ks

The protein-coding nucleotide sequences were first translated into amino acid sequences and aligned. Then, this alignment was used to define the alignment of the corresponding nucleotide sequences to avoid frame-shifting indels as alignment artifacts [46]. Ka and Ks were estimated in two ways: i) by using the PBL method [47], [48] implemented in DAMBE [46], [49], and ii) by the likelihood-based method implemented in the YN00 program in the PAML package with the resulting Ka and Ks designated by dN and dS, respectively in table 1 [50].

### pI estimation and outlier detection

We have investigated whether the pI of presumably orthologous proteins were similar. In order to test this, we gathered the protein sequences of the orthologs of ERG2, 6, 24, 25, 26 and 28 listed in the HomoloGene division of the NCBI database and estimated their pI (http://www.expasy.ch/tools/pi_tool.html). Then, we asked whether the corresponding *Drosophila* and *Caenorhabditis* sequences displayed outlier pI values. In short, we performed an extreme studentized deviate test to determine whether one of the values in the pI list was a significant outlier from the rest.
